# How light is too light touch: The effect of a short training-based intervention on household poultry production in Burkina Faso^☆^

**DOI:** 10.1016/j.jdeveco.2021.102776

**Published:** 2022-03

**Authors:** Jessica Leight, Josué Awonon, Abdoulaye Pedehombga, Rasmané Ganaba, Aulo Gelli

**Affiliations:** a International Food Policy Research Institute, United States of America; b Tufts University, United States of America; c AfricSanté, Burkina Faso

**Keywords:** Training, Livestock production, Sub-Saharan Africa, Randomized controlled trial

## Abstract

This paper reports on the effects of a training-based intervention seeking to increase household engagement in poultry production in Burkina Faso, analyzing data from a large-scale cluster randomized trial in which 1798 households in 60 communes were observed over a period of three years. The intervention SELEVER — entailing a short series of trainings for households as well as capacity building for local animal health and credit services — had little effect on household poultry production and no effect on profits. There is some evidence of an increase in the utilization of poultry inputs and an associated reduction in poultry mortality, primarily for larger poultry producers; however, there is no evidence of any treatment effects for the smallest producers.

## Highlights

• A short training-based intervention seeking to increase household engagement in poultry production in Burkina Faso was not effective in increasing poultry flock size or revenue.
• Larger producers did show evidence of a shift in poultry practices, and a higher level of poultry knowledge.
• However, smaller and more subsistence-oriented producers were largely unresponsive.

## 1 Introduction

Livestock production is widely viewed as a promising avenue for poor households in rural sub-Saharan Africa to diversify beyond staple crop production and enhance their economic welfare, and this is particularly true for poultry production given its relatively modest investment requirements (Sonaiya and Swan, 2004). In the Sahel, household engagement in poultry raising is extremely common: in Demographic and Health Survey data, nearly 60% of households in the region own poultry (Headey and Hirvonen, 2015).

At the same time, substantial evidence suggests that especially given the prevalence of small-scale, backyard production in contexts such as Burkina Faso (analyzed here), there is a substantial gap between the low use of basic production technologies and recommended best practices. Low adoption of modern inputs may be associated with high levels of mortality — widely documented for the poultry sector — and the persistence of suboptimally low production scale (Richard, 2007, Somé, 2015). A large literature in development economics has analyzed the effects of business training in targeting similar deficiencies in knowledge and practices among entrepreneurs (particularly small entrepreneurs) in the developing world, and a recent review and meta-analysis suggested positive effects of such training programs (McKenzie, 2020). However, this literature does not include any papers analyzing training targeted to the livestock sector, and a parallel literature analyzing agricultural training programs (described in more detail below) has generally produced more pessimistic findings.

This paper provides new evidence on the effectiveness of training for the livestock sector, reporting on a randomized controlled trial implemented in Burkina Faso evaluating SELEVER (Soutenir l’Exploitation Familiale pour Lancer l’Élevage des Volailles et Valoriser l’Économie Rurale), a multifaceted intervention seeking to increase poultry production and improve the nutritional status of women and children among poor rural households (Gelli et al., 2017). SELEVER is delivered by a non-governmental organization Tanager and includes three key elements: training for producer households, training and enhanced provision of equipment to village-level providers of vaccination and extension services, and enhanced access to credit.^2^ Importantly, there is no transfer of assets or provision of subsidized or free inputs.

The intervention was designed in the context of distinct challenges faced by two sets of household producers within the Burkinabe poultry sector. First, very small producers (defined as those characterized by flocks of fewer than 20 birds and especially fewer than five birds) own poultry only as a buffer stock asset, and do not seek to commercialize poultry systematically. For these households, the intervention provides information about the benefits of commercialization and thus induces households to shift their behavior toward engagement in poultry production as an income-generating activity. Second, larger producers have more substantial flocks and commercialize more readily, but they are characterized by low use of modern inputs (poultry feed, vaccinations, and modern housing methods) and high mortality, constraining their ability to expand their flock and increase their sales (Somé, 2015). For these producers, the intervention provides education about modern production practices in order to target these constraints.

The analysis measures the effects of SELEVER on household poultry production following two and a half years of program implementation, analyzing a sample of 1,798 households observed in 119 villages in 60 communes across three regions. Within the original sample, 1669 were surveyed again at endline between March and August 2020, for a minimal attrition rate of 7%. (Due to disruptions in fieldwork linked to the ongoing COVID-19 pandemic, data collection was implemented in two phases, launching first in March and then resuming subsequently in June.) The primary analysis is an intention-to-treat specification: pre-specified primary outcomes include poultry production, sales and profits, and pre-specified secondary outcomes include poultry-related knowledge, use of poultry inputs, credit access and utilization, marketing practices, and poultry mortality (Gelli et al., 2017).

Our primary findings suggest that there are some effects of the intervention in the first steps of the causal chain: there is a significant increase in poultry-related knowledge, and households are more likely to feed poultry concentrate feed and vaccinate them. However, the shifts in input usage appear to be attenuating rapidly over time as exposure to the program declines; we can identify this shift by exploiting an additional, smaller-scale survey round conducted in fall 2019, approximately three months prior to the closure of the main intervention activities.

Despite this pattern of increased input use, however, there are no statistically significant increases for the primary outcomes, stock value, revenue and profit. In fact, point estimates are small in magnitude or negative. There is some evidence of an expansion in the number of mature poultry owned, suggesting an increase of 14% relative to the control arm, and an associated reduction in poultry mortality. While consistent with the increased use of vaccinations and other poultry inputs, this effect is only marginally significant.

When we further disaggregate the intervention effects to analyze the impacts for both small and large producers (defined using a pre-specified cutoff of twenty birds), we find that in general, the experimental effects seem to be concentrated among large producers. The estimated treatment effects for small producers are null or negative, while the increase in input use (and the weak reduction in mortality) is observed for large producers. This suggests that the intervention was not at all effective in inducing households who do not engage in systematic commercialization of poultry to shift their production-related behaviors. For households who are so engaged, there was some shift in inputs, but the returns to these inputs do not seem to be particularly high; nor did their use lead to any additional poultry sales.

Along with the broader literature about business training summarized in McKenzie (2020), this paper joins a more recent experimental literature analyzing the effects of agricultural extension or training programs, predominantly for crop cultivation. The literature on agricultural extension programs is large, and an earlier wave of largely (non-experimental) papers is well-summarized in Anderson and Feder (2007). More recently, Cole and Fernando (2020) analyze a digital form of extension service provided to farmers in India and find evidence of increases in knowledge and input use (though these estimates are not robust to multiple hypothesis testing), and null effects on yield and profits. Fabregas et al. (2019) analyze a series of randomized trials of SMS-based informational interventions targeting farmers in sub-Saharan Africa and find effects on knowledge and practices that do not persist, though they do not analyze yields or profits; similarly, Casaburi et al. (2019) also find null effects of agricultural extension on yields among sugar cane contract farmers in Kenya, though they have no data on farm practices. Fafchamps and Minten (2012) find minimal effects on practices (price arbitrage and grading) of a digital price information service offered to farmers in India and no effect on economic outcomes such as profits. A non-experimental evaluation in Malawi also finds no effect of farm extension on average, though possibly more effects for farmers who report the information to be useful (Ragasa and Mazunda, 2018).

Another set of recent papers has analyzed the effectiveness of “farmer field days” providing demonstrations and information to farmers about new agricultural technology. In India, this strategy was highly effective in stimulating adoption of a new seed, use of which has been shown to reduce downside risk and crowd in other productive agricultural investments (Emerick et al., 2016, Emerick and Dar, 2021). However, other evaluations in Kenya and Malawi found that field days largely did not succeed in encouraging farmers to adopt new soil management techniques (Fabregas et al., 2017, Maertens et al., 2021).

Broadly, our findings are consistent with the more pessimistic strain of the above literature: we see some evidence of an effect of training on production practices, but suggestive evidence these shifts are time-limited and thus fading out, and no effects on economic outcomes. This is true even for a sample of relatively experienced chicken producers, as was observed for relatively experienced (though not necessarily highly commercial) cotton farmers in Cole and Fernando (2020). Existing evidence in the literature around training for livestock itself is very limited. In Rwanda, a program providing cows as an asset transfer was differentially more effective when training was also provided, though the offer of training was not randomized (Argent et al., 2014), and in Nepal, a training-only program targeting goat production increased financial inclusion and women’s empowerment, though there were no effects on assets, expenditure or food security (Janzen et al., 2018).

In addition, a growing interdisciplinary literature has analyzed the effects of nutrition-sensitive agricultural development programs such as this intervention on a range of outcomes including food availability, access to food, diet quality, women’s empowerment, and gender norms (Ruel et al., 2018, Heckert et al., 2019, Olney et al., 2015, Olney et al., 2016).^3^ However, rigorous evidence on the role of livestock market-based interventions in improving smallholder incomes — as distinct from nutritional and consumption outcomes — is very limited (Ruel et al., 2018, Ruel and Alderman, 2013). This paper is one of the first to our knowledge to provide evidence of effects on household production and income, and suggests that at least in this context, a light-touch, training-based intervention was not sufficient to generate meaningful effects on economic welfare.

## 2 Experimental design

### 2.1 Context

Poultry production is a major contributor to the agricultural economy of Burkina Faso, and it remains an important economic activity for large numbers of rural households (Gning, 2005). Data from the 2010 Agricultural Census shows that 99% of poultry producers are smallholders (defined as owning a flock of between five and 50 poultry), owning 98% of the total flock, and producing 99% of the supply of meat and 86% of the supply of eggs (Food and Agriculture Organization, 2018). Similarly, another more recent household survey suggests that 80% of rural households own poultry (Gelli et al., 2019). In addition to its salience for the rural economy, poultry production has important implications for the economic role of women, who often manage the daily care of poultry, though men are generally responsible for sales (Eissler et al., 2020).

Despite the widespread prevalence of poultry raising in Burkina, there is substantial heterogeneity in households’ engagement in the sector. A recent report analyzing the poultry sector identified multiple segments of production and noted that households owning the smallest flocks (fewer than 20 birds, and especially fewer than five birds) are largely subsistence producers characterized by minimal investment in inputs and (particularly for the smallest farmers) limited interest in commercialization or flock expansion (Somé, 2015). These households do not regard poultry as meaningful source of income, but rather use the flock as a buffer stock, selling poultry in case of an unexpected expense as a form of insurance, or when they have a requirement for substantial liquidity (Hoffmann et al., 2020, Zougouri and Cook-Lundgren, 2016). Own-consumption of poultry is also minimal for these households, given that the implicit cost of this consumption is very high (Abioye and Adegoke, 2016).

Larger producers (identified in this report as flocks of more than 20 birds) are by contrast more oriented toward poultry commercialization, or at least open to expanding the scale of production and identifying commercialization opportunities, and more likely to use modern inputs. In the succeeding analysis, we will also explore the characteristics of these producer segments in our own sample.

### 2.2 Intervention

The SELEVER intervention is multifaceted, encompassing intervention components in three broad sectoral areas: poultry, nutrition and health, and gender (Gelli et al., 2017). This analysis will focus solely on the poultry component; results analyzing the effects of nutrition/health and gender interventions will be reported separately. The SELEVER poultry intervention centered around the provision of training on poultry production to rural households constituted in village-level producers’ and savings groups. Eight poultry training modules were delivered by trained facilitators (similar to extension agents) to the producers’ groups in up to eight sessions. While the precise mechanism of delivery varied locally, a process evaluation conducted by the research team suggested that frequent modes of delivery included initial workshops of longer duration (two to three days) in conjunction with more regular training sessions, delivered by screening video training modules, that were offered at producer associations’ regularly scheduled (approximately monthly) meetings (Gelli et al., 2019).

The eight modules focused on the following topics: opportunities for income in poultry production; appropriate housing practices; poultry reproduction; chick management; best feeding practices; compiling poultry feed; poultry health; and business and entrepreneurial management for poultry production. Key specific lessons that were highlighted included the importance of production practices such as confining poultry in chicken coops, providing them with diversified sources of feed (rather than allowing them to scavenge), and vaccinating and deworming them regularly. All of these strategies were argued to reduce poultry mortality, and thus enable households to stabilize and expand their poultry flocks. The importance of maintaining a hygienic environment by removing chicken feces regularly was also emphasized. While the trainings were intended to also provide some information about commercialization opportunities and potential earnings from poultry production, effectively this content was de-emphasized relative to the information around poultry practices.

In addition, SELEVER sought to strengthen village animal health and credit services. Rural poultry producers rely on a system of Village Volunteer Extension Services Workers (VVVs following the name in French), a volunteer corps trained by government staff to provide basic livestock services (e.g., vaccinations, deworming and nutrition advice).^4^ The government also bulk purchases vaccines and sells them at discounted rates to VVVs, who can then earn income on the provision of these services; they are not constrained to offer free or low-cost inputs (Gning, 2005). Importantly, VVVs are active across the sample in both treatment and control arms, and they are not an innovation introduced by this intervention. However, SELEVER provided additional training to the VVVs as well as a start-up kit including a cold storage unit, a syringe and needle, and 100 doses each of poultry vaccines and deworming pills. The program also developed a cohort of women VVVs to better reach women poultry producers.

Finally, in the credit sphere, two microcredit organizations collaborated with Tanager to expand into the treatment communes, providing at least one microcredit branch in each commune if such a branch was not already present. They also assumed the role of providing credit to poultry producers participating in SELEVER, but there was no explicit guarantee. Credit was available based on standard commercial criteria designed to identify a viable enterprise, and these criteria were largely discretionary.

If we frame the intervention in terms of the barriers to poultry commercialization faced by target households, it is clear that these barriers are somewhat distinct comparing across the two sets of households described above. For households that are smaller, subsistence-only producers, the first and most important characteristic identified by the program designers is that these households do not view poultry production as a meaningful activity to generate income, but rather a method to maintain a buffer stock against income shocks. This may be due to an absence of information about the returns to poultry production; limited access to markets; and/or credit constraints that prevent expansion of production. In targeting these households, the intervention essentially provided information (via the training modules) about the potential benefits of expanding poultry production in order to convince households to attempt more commercialization of poultry. (They could, of course, also benefit from the more specific education provided in refining their poultry practices.)

For households that are larger producers, by contrast, the key barriers identified were low use of modern inputs (vaccinations, modern feed, improved poultry coops, etc.), associated with an extremely high level of poultry mortality that renders it challenging to expand poultry flocks.^5^ The intervention sought to target these barriers by providing information, capacity building to input provision systems (the VVVs), and credit. One key point to note is that there was no strong evidence of demand-side barriers (i.e., low demand for chickens leading to low prices), and thus SELEVER did not target any such barriers (Somé, 2015). This point was summarized in the original study protocol: producers face “multiple constraints in responding to demand from actors further downstream (e.g. retailers, processors)” and SELEVER seeks to “alleviate these constraints (e.g. increase poultry flock size through reduced mortality from improved uptake of vaccinations and other poultry management practices)”. While the intervention was not conceptualized as exclusively targeting larger producers, in implementation it did primarily target barriers relevant for these producers; this was also the primary reason that large producers were oversampled in the evaluation design.

**Fig. 1:**
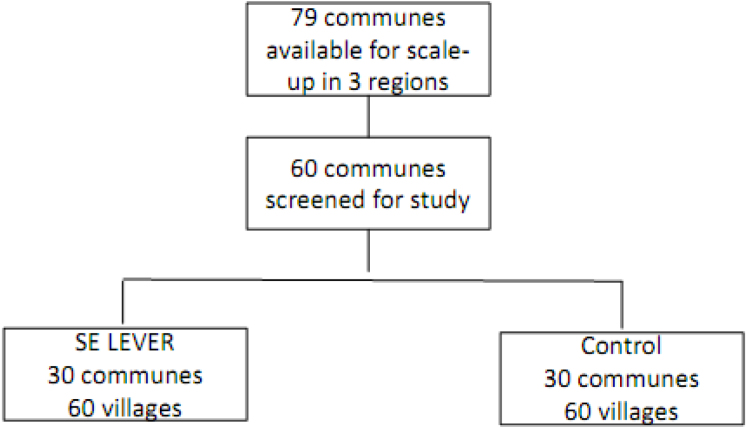
Evaluation design.

### 2.3 Study design

The sample for the evaluation included 60 communes (rural and peri-urban) within three targeted regions of Burkina Faso (Boucle du Mouhoun, Centre-Ouest, and Hauts-Bassins). These communes were selected randomly from a group of 79 communes in these regions identified as eligible for scale-up based on the following criteria: they had not previously been exposed to SELEVER pilot programming, they were designated as rural or peri-urban in the national census, and they were accessible by road year round. The randomization design is summarized graphically in Fig. 1, assigning 30 communes to intervention and 30 communes to control using a re-randomization procedure described in more detail in the trial protocol (Gelli et al., 2017).^6^ Two villages were selected within each commune (the commune is the higher level of administration).^7^ In sampled communities, households with a woman 15–35 years of age and a child aged 2–4 years living together were eligible for inclusion. A full household census was conducted to identify eligible households, and 15 households were then randomly selected for inclusion, with oversampling of large poultry producers (flock size of more than 20 mature birds). This yields a target baseline sample of 1800 households (60 communes, 120 villages). Ultimately, data was collected in only 119 villages; one village was omitted due to a failure to correctly identify the community in the field.

As this trial is multidisciplinary, a full trial protocol was published (Gelli et al., 2017) that specified primary and secondary outcomes, and we utilize this protocol as our reference rather than a separate pre-analysis plan. Pre-specified primary outcomes include poultry production, sales and profits, and pre-specified secondary outcomes include women’s empowerment, poultry-related knowledge, use of poultry inputs, credit access and utilization, marketing practices, and poultry mortality.^8^ Power calculations were conducted for the primary outcomes using data from an earlier evaluation (Olney et al., 2015), and indicate the analysis has 80% power to detect an effect size of .18 standard deviations for household poultry production and .15 standard deviations for household poultry sales (Gelli et al., 2017).^9^ Ethical review and approval was provided by the Institutional Research Board at the International Food Policy Research Institute and the Comité Éthique pour la Recherche en Santé MS/MRSI in Burkina Faso.

**Table 1:** Summary statistics across arms: Baseline.

	(1)	(2)	(3)	(4)	(5)	(6)
	Control	Treatment	p-value	Small prod.	Large prod.	p-value
Age household head	43.534	44.286	0.032	40.687	45.748	0
Any primary education	.083	.059	0.009	6.988	9.882	0
(household head)						
Household size	8.791	8.87	0.084	.305	.573	0
Polygynous	.467	.484	0.081	.079	.067	.144
Any poultry	.957	.949	0.539	.88	.995	0
Stock value	91.573	94.991	0.110	24.277	132.768	0
Flock size	50.549	38.004	.305	10.219	63.846	0
Number mature birds	29.461	24.302	0.776	6.324	38.68	0
Number mature birds	23.849	21.743	.585	5.398	32.768	0
(male ownership)						
Number mature birds	4.428	1.566	.396	.643	4.361	.133
(female ownership)						
Number consumed	4.886	3.943	.687	1.275	6.218	0
Poultry mortality ratio	1.683	1.424	.225	2.734	1.018	.002
Any poultry revenue	.654	.651	.423	.418	.787	0
Number sold	9.252	10.123	.466	2.711	13.677	0
Poultry revenue	63.181	72.556	.152	20.187	95.113	0
Poultry profits	49.653	55.271	.534	14.124	74.378	0
Reports sales at home	.272	.329	.106	.204	.356	0
Reports sales at market	.277	.241	.846	.123	.337	0
Reports sales door-to-door	.059	.054	.188	.038	.068	.05
Any egg revenue	.049	.027	0.239	.005	.056	0
Egg revenue	.275	.136	0.553	.019	.314	0
						
Joint-test p-value	0.029					
Joint-test p-value	0.536					
(poultry variables)						

Note: This table reports key indicators of interest corresponding to household demographics and poultry production as observed at baseline in communes assigned to the control and treatment arms, and for large and small poultry producers. Any poultry, any poultry revenue, any egg revenue, and reports sales are binary variables; stock value, revenue, profits, and egg revenue are reported in dollars; number of mature birds and flock size are reported in units of birds; the poultry mortality ratio is defined as the number of birds reported lost to illness over the preceding six months divided by the flock size as of the survey date. Column (3) reports the p-value corresponding to a regression in which the indicator of interest at baseline is regressed on a binary variable for assignment to SELEVER; the regression is weighted to take into account baseline sampling probabilities, and standard errors are clustered at the commune level. At the bottom of the table we report a joint p-value corresponding to a F test across all outcomes analyzed.

### 2.4 Data collection

**Fig. 2:**
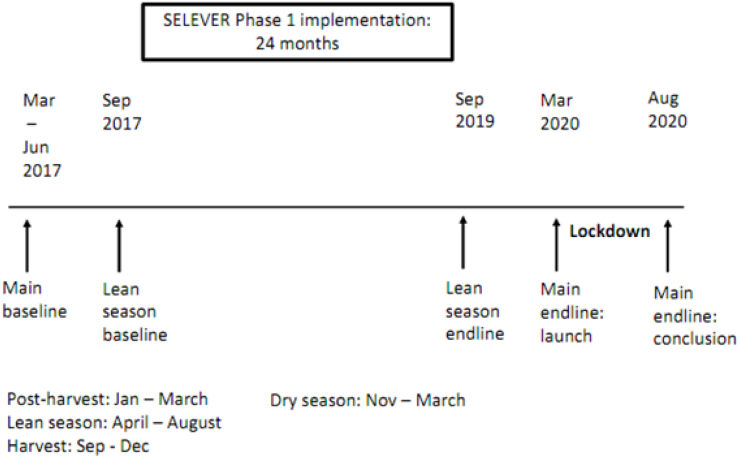
Timeline.

Fig. 2 summarizes the key timeline relevant for this project. The main baseline survey was conducted between March and June 2017, and the main endline survey was launched in March 2020; due to interruptions linked to the COVID-19 pandemic, data collection was conducted in March and then resumed in June following the conclusion of pandemic-related lockdowns in Burkina Faso, concluding in August 2020. In addition, two supplemental surveys were conducted in an alternate season (the lean season, September) in 2017 and 2019. This analysis will primarily draw on the main endline survey, but some discussion of seasonal differences can be found below in Section 4. Implementation of the main intervention was rolled out in fall 2017 and continued for approximately 24 months, concluding around fall 2019.

In interpreting the timing vis-a-vis seasons of production, it is important to note that the cropping calendar is generally clearly delineated in Burkina Faso: major harvests are conducted in September to December, with January to March characterized as the post-harvest period. These patterns do not vary substantially with respect to the crops cultivated (in this sample, primarily sorghum, millet and corn). By contrast, April through August is the lean season (the period between planting and harvesting), in which food availability generally sharply contracts. Hence the secondary surveys conducted in September for this evaluation (and reporting retrospectively on the preceding six months) are denoted lean season surveys with respect to the crop calendar (Somé, 2018).

For chicken production, there is no pronounced seasonality of production, other than a spike in demand linked to major festivals; however, there some seasonality in mortality risk, particularly risk linked to Newcastle disease. This risk is generally noted to be higher in the dry season: approximately November to March, during the harvest and post-harvest period (Sonaiya and Swan, 2004).

In each wave of data collection, household surveys were conducted, collecting sex-disaggregated data on poultry production (including inputs, flock size, marketing, revenue, and profits). The first survey respondent for the poultry module was the male member of the household identified to be most knowledgeable about the household’s poultry activities (usually the head of household), and the second survey respondent was the female member identified as most knowledgeable about poultry production among the women of the household. Each respondent reported separately the number of poultry owned by type of bird (roosters, hens, pullets, and chicks) and breed (chickens and guinea fowl), and answered a series of detailed questions about poultry inputs, sales, and mortality. The reference period for the variables of interest is generally the previous six months. The respondent reported whether there was any engagement in poultry production over this period, and for poultry practices reported whether she or he engaged in that practice over this period. For variables such as input cost, revenue, and profits, the respondent reported the total amount spent or earned over this period. The only exception is flock size and the estimated value of the flock, reported as of the day of the survey.

**Table 2:** Summary statistics for SELEVER exposure in treatment communities.

	(1)	(2)	(3)	(4)	(5)	(6)	(7)	(8)	(9)	(10)	(11)
	Primary (post-harvest) endline, 2020							Lean season endline, 2019			
	Control arm			Treatment arm				Control arm		Treatment arm	
Indicator	Mean	St. dev.	Obs.	Mean	St. dev.	Obs.	p-value	Mean	Obs.	Mean	Obs.
Household reports benefits	.01	.1	827	.13	.34	815	0.000	.02	346	.27	664
from SELEVER											
Any member attended poultry training	.07	.25	827	.27	.44	815	0.000	.12	346	.49	664
Attendance at poultry training: female	.03	.18	802	.18	.38	787	0.000	.06	313	.37	637
Attendance at poultry training: male	.06	.23	716	.21	.41	704	0.000	.09	298	.43	564
Number of trainings attended	1.23	1.43	53	1.83	1.76	215	0.006	1.2	38	2.63	313
(all members)											
Any member attended business training	.07	.25	827	.17	.37	815	0.000	.11	346	.29	664
Attendance at business training: female	.04	.19	802	.1	.3	787	0.000	.08	313	.21	637
Attendance at business training: male	.04	.21	716	.12	.32	704	0.000	.05	298	.21	564
Any member participates in producers’ group	.02	.14	827	.15	.36	815	0.000	.04	346	.24	664
Participation in group: female	.01	.09	802	.09	.29	787	0.000	.02	313	.17	637
Participation in group: male	.02	.14	716	.13	.33	704	0.000	.03	298	.2	564
Any member knows VVV	.9	.3	827	.95	.23	815	0.004	.67	346	.81	664
Ever received VVV services	.54	.5	802	.59	.49	787	0.137	.47	313	.53	637
											
Any reported exposure											
to SELEVER programming	.12	.33	827	.4	.49	815	0.000	.2	346	.57	664

Note: This table reports summary statistics (mean, standard deviation and number of observations) for variables capturing program exposure as observed in the post-harvest endline for individuals in the control and treatment arms in Columns (1) through (6). Column (7) reports the p-value corresponding to a regression in which the indicator of interest is regressed on a binary variable for assignment to SELEVER; the regression is weighted to take into account baseline sampling probabilities, and standard errors are clustered at the commune level. Columns (8) through (11) report the mean and number of observations for variables capturing program exposure in the lean season endline conducted in 2019.

## 3 Empirical results

### 3.1 Characterizing the sample

Table 1 presents baseline summary statistics and p-values corresponding to tests of balance across experimental arms.^10^ The table includes demographic characteristics and baseline poultry production, and all monetary values are presented in real 2017 U.S. dollars. The average household includes nine members and is led by a male head of around 45 years of age. Only 7% of household heads have received some primary education, and about half of households are polygynous.^11^ Virtually all households are engaged in poultry production, reporting a flock of around 44 birds total (29 mature birds) at baseline.^12^ Approximately 80% of birds owned are designated as owned by the male, while only 20% are owned by women. The poultry mortality ratio (defined as the ratio of poultry reported lost to illness over the preceding six months to flock size as of the day of the survey) is extremely high, more than 1.5 on average, suggestive of significant mortality risks.

Baseline revenue is calculated as the sum of cash revenue earned from poultry sales over the preceding six months in conjunction with the imputed value of own-consumption and is estimated to be around $68. Baseline profits (calculated as the difference between this poultry measure and reported explicit input costs) are around $52.^13^

The p-values for the tests of balance are reported in Column (3) of Table 1, and generally suggest that the hypothesis that covariates are balanced across arms cannot be rejected for poultry-related variables. For demographic variables, there are some differences that are significant: households in the treatment arm are characterized by heads who are slightly older and less likely to be educated, and households in treatment communities are more likely to be polygynous. (The differences in age and the probability of polygyny are small in magnitude though precisely estimated, while the difference in the probability of any education is relatively large.) The table also reports the p-value corresponding to a joint F-test of balance across all covariates reported. The hypothesis of balance can be rejected for the full set of covariates (p=0.029), but not for the set of covariates linked to poultry production (p=0.536).

In addition, in characterizing the landscape of poultry production in Burkina Faso and in our main analysis of treatment effects we draw on the distinction between small or subsistence-only producers and large or commercialization-oriented producers. As previously noted, the sample purposively oversampled large producers, identified as households reporting a flock of 20 or more chickens, in order to facilitate the analysis of heterogeneous effects for households reporting flocks of various sizes. More specifically, we construct a mean estimated flock size equal to the mean of three available pre-intervention observations (flock size in census, post-harvest baseline, and lean season baseline). We then generate a binary variable for large producer equal to one if this mean flock size is over 20, and Columns (4) and (5) of Table 1 present baseline characteristics for these two groups of producers, as well as a p-value test of equality.^14^

It is evident that large producers are meaningfully different. Small producers have on average only 10 birds (of which six are mature), compared to 64 (almost 40) for large producers. The number consumed over the last six months is around one for small producers, but six (i.e., approximately one chicken monthly) for large producers. The mortality ratio (defined as the number poultry reported lost to illness over the past six months relative to flock size as of the day of the survey) is extremely high on average, and also significantly higher for small producers (2.7) compared to large producers (1.0).^15^

**Table 3:**
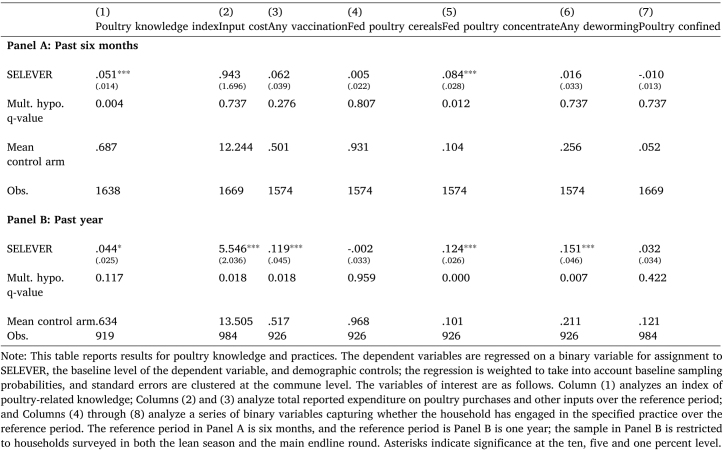
Effect of SELEVER on poultry knowledge and practices.

**Table 4:**
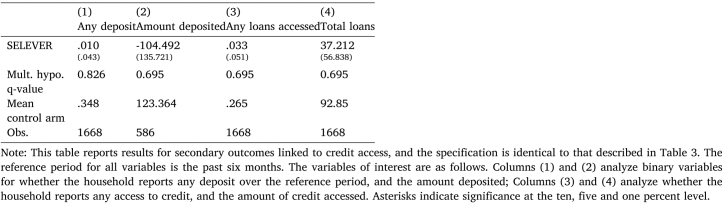
Effect of SELEVER on credit access.

Moving on to commercialization-related behaviors, less than half of small producers sell any poultry, while nearly all large producers do, and the number sold, revenue and profits are around five times larger for large producers. (Profits from poultry production also constitute around 15% of estimated total household expenditure over the reference period of the previous six months for large producers, compared to only 4% for small producers; this summary statistic is not noted in the table for concision.) In addition to these differences in the level of sales, we can also identify that — consistent with the prior literature — small producers sell at least partially in response to adverse household shocks, or increased demand for lump sum expenses (e.g., school fees). Table A1 shows that at baseline, small producers exhibit a significant correlation between revenue from poultry production and the probability of reporting any shock over the previous six months, as well as the school enrollment rate of children under 18 in the household; these correlations are zero or negative and not statistically significant for large producers. This is consistent with the hypothesis that small producers are not primarily engaged in commercialization of poultry for income purposes, but rather using poultry as a source of occasional liquidity.

While we do not have large-scale data on markets in which the households participate, households do report whether they have sold at home (to traders who travel door to door), at markets, or at home over the reference period. It is evident in Table 1 that sales at home are roughly as common as sales at markets, on average, suggestive of low spatial integration in sales across the evaluation region.^16^

**Table 5:**
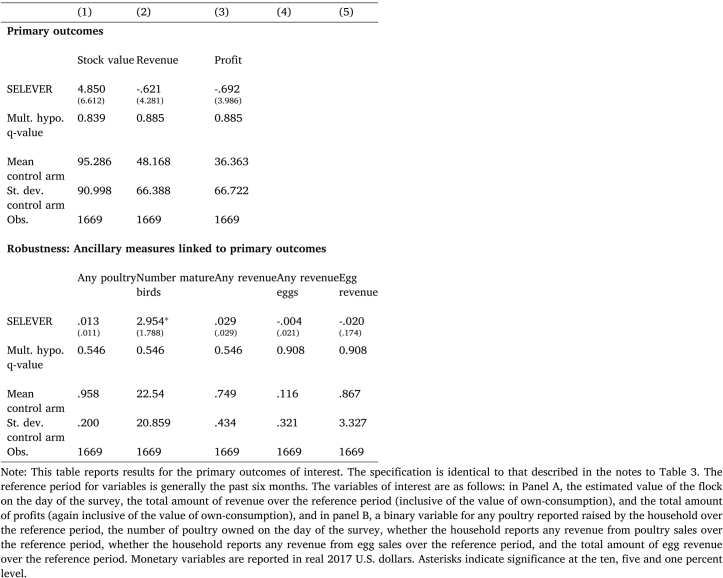
Effect of SELEVER on household poultry production: Primary outcomes.

**Table 6:**
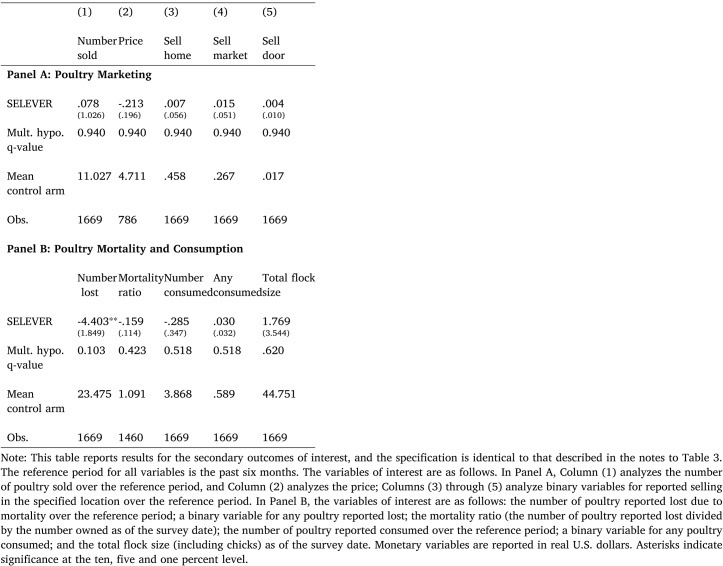
Effect of SELEVER on poultry marketing, mortality and consumption.

### 3.2 Intervention exposure

In order to assess households’ engagement with the SELEVER intervention in the communities in which it was implemented, Table 2 reports a series of variables linked to exposure; Column (7) reports a p-value corresponding to a test of equality across treatment and control arms.^17^ In addition, for comparison we report in Columns (8) through (11) parallel variables as observed within the longitudinal subsample surveyed in the lean season endline conducted in 2019. Intervention activities largely concluded at the end of 2019, and thus some decline in reported exposure in the 2020 survey is expected. (The time period for reported exposure is over the previous 12 months; thus in 2019, households report on exposure between roughly September 2018 and September 2019, while in 2020, households report on exposure between roughly July 2019 and July 2020, depending on when they were surveyed in 2020. Given the postponement of 2020 survey activities linked to the pandemic, the overlap between the recall periods of the two surveys is minimal.) Some persistent effects from intervention activities in 2019 may still be observed in poultry outcomes, and thus previous exposure is also useful to document.

On average, about 13% of all households in SELEVER communities report that they have benefited from the SELEVER intervention in the final endline survey in 2020, while 27% reported that they had benefited in the 2019 survey. (This question is a purely subjective perception of benefits, and can be equal to zero even for households who do report participation.) Nearly 30% reported that at least one member of the household had attended at least one SELEVER training, relative to about half of households reporting attendance in 2019. In both rounds, attendance is relatively balanced by gender, based on the numbers of households who report at least one woman attended and households who report at least one man attended. The average number of training sessions reported attended is 1.8 in 2020 and 2.6 in 2019, relative to eight total modules offered, though in some cases multiple modules may have been included in a single session. For business-oriented trainings, attendance is lower (17% in 2020 and 29% in 2019), but again balanced for men and women. Finally, participation in poultry producers’ groups is reported to be 15% in 2020, and 24% in 2019.

As noted above in the description of the intervention, VVVs are frontline government workers that are widely deployed across rural Burkina Faso; they are not an innovation specific to SELEVER. However, the intervention provided capacity building designed to strengthen the VVV system and bolster it with additional materials. Knowledge of the VVVs does appear to be increasing over time: in 2020, 90% of households in the control arm reported they know at least one VVV, rising to 95% in the treatment arm, while in 2019 the corresponding percentages ranged from 70 to 80%. Reported receipt of VVV services is slightly higher in treatment communities in both years (59% relative to 54% in 2020), but the difference is not statistically significant.

To summarize engagement with the intervention, we also report a variable capturing any exposure, equal to one if the household reports any benefits from SELEVER, attended a poultry training, attended a business training, or is a member of a producers’ group. (Knowledge of the VVV is excluded from this measure given that the VVV system is not an innovation introduced by the intervention.) Using this measure, around 40% of households in intervention communities reported some exposure to the poultry-related interventions in 2020, and 60% in 2019. There is also some evidence of contamination in the control arm, though the differences between the treatment and control arms are uniformly statistically significant: 12% of households in the control arm reported exposure to SELEVER in 2020, and 20% did so in 2019. This may reflect measurement error in which households mis-report their exposure to another, similar program.

### 3.3 Main specification

In order to analyze the experimental effects of interest, the primary specification is an ANCOVA regression in which outcome variables $Y_{ivct}$ for household i in village v, commune c and time period t are regressed on a dummy variable $S_{vc}$ capturing whether the commune is assigned to the treatment arm, as well as the baseline level of the outcome variable $Y_{ivc,t-1}$ and household covariates $\chi_{ivc,t-1}$.^18^

(1) $$Y_{ivct}=\beta_{1}S_{vc}+\beta_{2}Y_{ivc,t-1}+\chi_{ivc,t-1}+\varepsilon_{ivct}$$

Period t refers to the endline, and period t−1 refers to the baseline. Standard errors are clustered at the level of the commune, and the regression is weighted using the probability of selection to account for the over-sampling of large producers.^19^

In addition, for primary and secondary outcomes, we report q-values corrected for multiple hypothesis testing, using the Simes method (Newson, 2010). This correction is implemented for each family of secondary outcomes (Table 3, Table 4), for the three primary outcomes (Panel A of Table 5), and for ancillary variables linked to these primary outcomes (Panel B of Table 5).

**Table 7:**
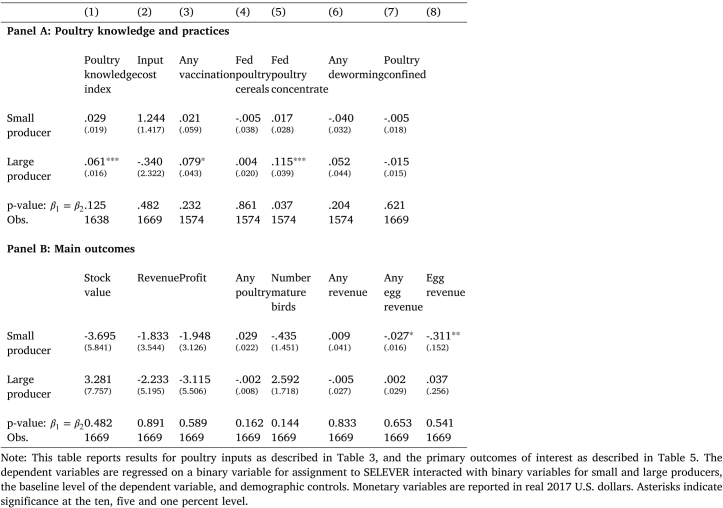
Heterogeneous effects.

### 3.4 Primary experimental effects

We focus first on outcomes that are proximally shifted by the intervention: in particular, poultry knowledge and use of poultry inputs, promoted by both the poultry training and the capacity building provided to VVVs, and access to credit and financing, promoted by the expansion of microcredit. These are secondary outcomes as identified in the original protocol. We then present the results for the primary outcomes, which have a clearer interpretation as indices of welfare: the scale of chicken production, revenue and profits.

Table 3 reports the estimated treatment effects for poultry practices. In Panel A, it is evident that households in treatment communities are more knowledgeable about poultry production (an increase of .05 in a knowledge index relative to a control mean of .69; the index corresponds to the percentage of nine questions for which the respondent provided correct answers). They are more likely to report vaccinating their poultry over the previous six months (though this effect is not significant at conventional levels), and are more likely to use concentrate feeds. The effects on knowledge and feeding practices are significant even when adjusted for multiple hypothesis testing.

In Panel B, we analyze the same variables reported over a reference period of the past year, using additional reports provided by a random subsample of households that were also surveyed approximately six months prior to the endline survey during the lean season survey. The dependent variables are constructed to be the mean across rounds for the knowledge index and input cost, and a binary variable equal to one if a household reports use in either round for the input use variables. Here, we see evidence of an increase in knowledge that is of roughly consistent magnitude; some evidence of an increase in total input cost for poultry ($4.43, relative to a mean in the control arm of $13.51); and a significant increase in the probability of any vaccination, feeding poultry concentrate, and reporting any deworming. These effects are generally statistically significant when employing the q-values as well. The pattern suggests that there has been some meaningful shift in the use of poultry inputs during the intervention period, but also attenuation over the year preceding the endline survey — consistent with the evidence previously presented of a substantial reduction in program exposure between 2019 and 2020. As the intensity of interaction with SELEVER has diminished, it seems that farmers have increasingly dropped their use of new production practices, and hence effects are reduced in the preceding six months.

It may also be useful to decompose the effects on knowledge: the knowledge index is constructed as the sum of responses to nine questions, of which three pertain to poultry feeding, and six to poultry hygiene. If we construct separate knowledge indices for these two domains, we observe a positive effect on both, but a larger effect on hygiene-related knowledge (the effect on poultry feeding knowledge is narrowly insignificant at the ten percent level). This is broadly consistent with the observed shift in practices focusing on vaccination, use of poultry concentrates for feeding, and deworming: some shift across both domains, but a particular shift in hygiene-related behaviors.

Next, Table 4 reports outcomes linked to microcredit. There is no evidence of any treatment effects, suggesting that the intervention did not effectively shift access to financing despite identifying this as a goal. The criteria identified for financing may have been too restrictive for many households. Alternatively, there may have been a parallel expansion of credit facilities in the control arm.

Given these effects on the initial steps in the causal chain, Table 5 reports the effects for the primary trial outcomes. In Panel A, it is evident that there is no significant effect on reported stock value, revenue or profits for poultry production, and the latter point estimates are in fact negative and close to zero. In Panel B, there is no evidence of any significant effects on the probability of reporting poultry production, the probability of reporting revenue from poultry production, the probability of reporting revenue from egg production, or the amount of revenue from eggs. There is an increase in the reported number of mature birds owned that is significant at the ten percent level in the conventional specification, though not statistically significant when corrected for multiple hypothesis testing. The magnitude suggests an increase of three birds relative to a mean in the control arm of 22.^20^

Table 6 then presents additional results on variables linked to marketing practices (in Panel A) and poultry mortality and consumption (in Panel B) that allows us to unpack the channels for these effects. In Panel A, there is no evidence of any shift in the number of birds sold and the price, or the marketing location, consistent with the observed null effects on revenue. In Panel B of Table 6, there is some evidence of a decline in the number of poultry lost to disease, consistent with the previously reported increase in total poultry owned. The coefficient suggests a reduction in total losses of four and half poultry relative to a mean in the control arm of 33; it is significant at the five percent level using conventional standard errors, and close to significant at the ten percent level using multiple hypothesis-adjusted standard errors (p=0.123).^21^

Finally, in order to corroborate the null effects observed for revenue and profits from poultry production, we also report treatment effects for total household expenditure per-capita as well as expenditure on food and non-food items. These results are reported in Panel A of Table A3 in the Appendix, and consistent with the previously presented evidence there are no significant effects on household expenditure. We also estimate treatment effects for some simple summary measures of crop cultivation (total area cultivated in hectares, total production of crops in kilograms, and number of crops cultivated) to assess whether there are any ancillary effects of the intervention on households’ other economic activities. These results are presented in Panel B of the same table, and again show no significant effects.

We also explored heterogeneous effects on the primary outcomes of interest for poultry owned by men, women and jointly. The evidence suggests there are substantial positive effects for production of poultry owned by women, counterbalanced by negative effects for poultry owned jointly (and weakly positive effects for poultry owned by men). This pattern may reflect a hypothesized “relabeling” in which exposure to the intervention leads women to identify previously jointly owned poultry as their own, rather than deferring to joint ownership. More details are provided in Section A1 in Appendix.

### 3.5 Heterogeneous effects for large producers

As previously described, the SELEVER intervention was designed to target two separate sets of barriers to expanded poultry production for two separate samples, small producers and large producers. In order to analyze these effects, we can re-estimate Eq. (1) including separate binary treatment variables for small and large producers, controlling separately for the large producer dummy. The results are presented in Table 7.

In Panel A, we examine poultry knowledge and practices. It is evident that the increase in poultry knowledge, vaccinations and the use of concentrate feeding is concentrated among large producers, while the comparable coefficients for small producers are small in magnitude or negative. Given the noise in the estimated coefficients, the hypothesis that the effects are equal cannot necessarily be rejected, though the difference is close to significant for the poultry knowledge index, and significant for the use of concentrate feeds.

Panel B reports the results for the main outcomes. Here, we generally observe null effects in the pooled specification other than a weakly significant increase in the number of mature birds: again, it is evident that this increase is primarily observed for larger producers (where it is narrowly insignificant), while small producers in fact show a decline in the number of mature birds. While this evidence should be interpreted cautiously given that the difference between the coefficients for the two subsamples is not always large, in general it is consistent with the hypothesis that the observed intervention effects reflect primarily a response by larger producers — who increase their knowledge around poultry production and shift practices, but with seemingly low returns — in conjunction with a true null response by small producers.

### 3.6 As-treated analysis

To conclude our empirical analysis, we also report an “as-treated analysis” analyzing the effects of SELEVER for those who in fact participated in the intervention, an analysis that was also pre-specified in the protocol (Gelli et al., 2017). Given that participation is non-random, this evidence should not be considered to be causal, and in general, if households who rationally expect higher benefits from SELEVER opt into participation, the as-treated effects would be expected to be more positive relative to the intent-to-treat effects.

First, in order to analyze selection into participating in treatment, we estimate a simple specification in which we regress various household-level measures of participation drawn from Table 2 on the same baseline covariates as reported in Table 1. The results are reported in Table A5 in Appendix. In general, households reporting any revenue from poultry production at baseline and reporting higher profits at baseline are more likely to participate in the intervention. (By contrast, those reporting revenue from egg sales are less likely to participate, possibly reflecting the fact that only the very largest producers report revenue from egg sales at baseline, and they may not perceive a benefit to additional services or information linked to poultry production.^22^)

Second, we estimate a difference-in-difference for households who do and do not participate in the intervention, employing kernel propensity score matching to compare households who endogenously opt in to SELEVER to similar households in the treatment arm (Heckman et al., 1998, Villa, 2016).^23^ These results are reported in Table A6; Panel A uses the pooled measure of exposure to the poultry-linked programming previously reported in Table 2, and Panel B uses a broader variable that is equal to one if households report exposure to poultry as well as nutrition programming provided by SELEVER. Using this second definition, mean exposure in the treatment communities is 25%. (The nutrition intervention and its effects will be described in further detail in a separate manuscript.)

The results in Panels A and B are consistent. Households who participated in SELEVER show evidence of an increase in the number of mature birds that is larger in magnitude and more precisely estimated relative to the estimated coefficient in the intent-to-treat specification (four when using the poultry-specific measure of exposure, and seven using the pooled measure, relative to a control mean of 23). There is also some weak evidence of an increase in the probability of reporting any poultry revenue, an increase of between six and 10 percentage points relative to a mean of 75% in the control arm. Again, however, there are no significant effects on stock value, revenue or profits.

## 4 Discussion

To sum up these findings, in general the evidence suggests that a relatively light-touch intervention providing poultry-related training to rural Burkinabe households did not have a significant effect in stimulating increased production of or profits from poultry. There is some weak evidence of enhanced use of poultry inputs and larger flocks; these effects are concentrated among households who were large producers at baseline, and also observed for households who participate more actively in the intervention.

To put these results in context, we will briefly summarize the findings from a previous analysis of treatment effects conducted using data collected in fall 2019, at the conclusion of the lean season (defined with reference to the cropping season as the period in which food availability substantially contracts). This was also a period in which intervention activities were still ongoing, as field activities concluded in late 2019. In the lean season data, there were substantial effects on the use of poultry inputs and thus on total input cost, as well as an increase in poultry revenue; the increases in cost and revenue were of roughly equal magnitude, however, yielding no increase in profits. There was also no reduction in poultry mortality (Leight et al., 2020).

The analysis in this paper uses the main endline survey conducted in 2020, by which point the intervention had concluded. The first obvious conclusion is that while the effect on poultry knowledge appears to be consistent, the shifts in poultry input practices are attenuating toward zero following the conclusion of SELEVER. In the absence of continued program activities, it seems that the behavioral shifts observed for poultry producers during the program period may not be persistent, a finding parallel to some evidence in the agricultural extension literature (Fabregas et al., 2019).

The second conclusion is that the returns to the increased use of inputs over both years appears to be low, other than a small reduction in mortality. The fact that the reduction in mortality is evident only in the main endline survey suggests that the returns to poultry vaccination are primarily realized in the dry season (November to March), a peak period of vulnerability to Newcastle disease, and thus are observed only in the spring 2020 survey whose recall period encompasses this period. The endline survey also collected data on past-month poultry mortality (as distinct from poultry mortality over the last six months, reported above) and this variable shows no significant effect. Since the past-month recall period would not overlap with the dry season for the majority of households, particularly those surveyed following the COVID-19 disruption, this again suggests that the reduction in mortality may be highly concentrated in the dry season period. The return to poultry vaccination is thus seasonal, but also seems to be small.

Returning to the design of the intervention vis-a-vis challenges faced by poultry producers in this context, SELEVER was clearly not effective in shifting the behavior of small producers who engage in poultry production primarily as a buffer stock economic activity, rather than a source of income. This suggests that the program may have been too weak a lever to convince households that to re-assess their perception of the economic potential of poultry production, and/or even if they absorbed some information, they faced other meaningful constraints. For larger producers, training and associated capacity building for VVVs induced some (temporary) shift in poultry vaccination and feeding, but the returns to the new practices do not appear to be large (though given the magnitude of the intervention effects, the analysis may lack statistical power to detect these returns). In the absence of any substantial increases in flock size, even large producers did not consume or sell any additional poultry, nor do they realize any associated income gains. That may be because the initial diagnosis — that low use of modern poultry practices was causing high levels of mortality and suboptimally low levels of poultry production and profits – was incorrect, or because the intervention was not powerful enough to generate a shift in practices large enough to have a detectable effect on poultry stocks.

These findings also link to a broader literature around economies of scale in poultry production. In richer countries, poultry production is overwhelmingly concentrated in high-input, large-scale producers that utilize modern breeds of poultry in conjunction with improved feeds, strict disease control mechanisms, and poultry housing (Narrod et al., 2008). In developing countries, however, the majority of production is “extensive” backyard production characterized by low inputs and low productivity (Mcleod et al., 2009, Gilbert et al., 2015). While there is no universal criterion for an intensive producer, the flock size necessary to begin to achieve meaningful economies of scale may be 100 birds or more (Beesabathuni et al., 2018); this corresponds roughly to the 98th percentile of the distribution of flock size observed in the pooled sample (treatment and control) in the endline sample analyzed here. Accordingly, another plausible interpretation of the findings is that rural Burkinabe households are simply producing at too small a scale to easily realize significant profits from poultry production, and that larger or more intensive interventions would be required to catalyze a shift toward significant economies of scale.

## 5 Conclusion

This paper seeks to provide evidence about the effectiveness of a relatively light-touch, informational intervention in expanding household poultry production in rural Burkina Faso. The SELEVER cluster randomized controlled trial tracked a large cohort of households over three years, including surveys in both major seasons. While there is some evidence that the intervention generated a shift in poultry practices, there is no robust evidence of substantial shifts in the number of poultry owned at the household level, revenue or profits. In this context, poultry training in conjunction with some enhanced availability of animal health and credit services does not seem to be sufficient to stimulate major transformation in the poultry sector or in the economic benefits households derive from it.

These findings also link to a recent literature around crop extension that has shown rather mixed results around the effects of extension on actual yield or profits for farmers, though some programs have positive effects on knowledge and practices (Cole and Fernando, 2020, Fabregas et al., 2019, Casaburi et al., 2019, Fafchamps and Minten, 2012). This is in contrast to the generally positive (though sometimes small) effects of business training in the developing world. One interesting direction for future research is to identify why the estimated effects of business training for non-agricultural entrepreneurs are so divergent when compared to the effects of training and informational interventions targeting agriculture and livestock producers, even (as in this sample) more experienced producers. Future work may explore whether targeting even larger producers who can realize greater economies of scale is a promising strategy for this type of intervention, though this targeting mechanism clearly has implications for the characteristics of households who benefit.

## CRediT authorship contribution statement

**Jessica Leight:** Investigation, Formal analysis, Writing – original draft, Writing - review & editing. **Josué Awonon:** Formal analysis, Writing – review & editing. **Abdoulaye Pedehombga:** Investigation, data curation. **Rasmané Ganaba:** Investigation, data curation. **Aulo Gelli:** Conceptualization, funding acquisition, investigation, Supervision, Writing – review & editing.

## Appendix A Supplementary data

MMC S1 On-line appendix for how light is too light touch.

## Footnotes

2 Additional dimensions of the SELEVER intervention not analyzed in this paper included behavioral change communication around nutrition and health designed to promote improved diets and nutritional status for women and children; and community-level sensitization around women’s economic empowerment.

3 A nutrition-sensitive agricultural intervention is defined as an intervention that seeks to “address underlying determinants of fetal and child nutrition and development— food security; adequate caregiving resources at the maternal, household and community levels; and access to health services and a safe and hygienic environment—and incorporate specific nutrition goals and actions”. See Ruel and Alderman (2013) for more details.

4 Until about ten years ago, the VVVs provided only vaccination services, and their name accordingly reflected this role (Vaccinateurs Volontaires Villageois). More recently, the V for “vaccinateurs” in French has been replaced by “vulgarisateurs” to denote their wider role in extension and information provision.

5 Estimates from the literature suggest that the gross mortality of chickens can reach 80 to 90% in Burkina Faso, though this is concentrated among chicks (Richard, 2007).

6 More specifically, a randomization routine that selected 30 treatment communes from the sampling frame, and two villages within each commune, was run with 3,000 replications. Villages that did not have the target population size for the intervention were excluded from the list. The research team then selected the randomization permutation in which the r-squared of a regression of village and commune covariates on assignment to treatment was minimized. An additional second-level randomization then assigned 50% of the communities in the SELEVER arm to receive an additional WASH (water, sanitation and hygiene) intervention focused on promoting community-led total sanitation concepts; there was a particular emphasis on the appropriate management of chicken waste.

7 There are 351 communes in Burkina, but a much larger number of villages.

8 Table 3 in the published trial protocol provides full details.

9 This is assuming an intracluster correlation of .001 at the level of the commune and between .05 and .1 at the level of the village. Data employed for power calculations was drawn from two observational studies evaluating food intake in the study regions, and a recent impact evaluation of the Helen Keller International homestead food production intervention (Arsenault et al., 2014, Martin-Prevel et al., 2016, Olney et al., 2016).

10 More specifically, baseline covariates are regressed on a treatment dummy, with standard errors clustered at the commune level. The regression is weighted to take into account household-level oversampling of large producers.

11 97% of household heads are male.

12 The available evidence generally suggests that the observed flock size in the pooled sample is broadly consistent with the observed flock size in nationally representative samples. Employing a rough back of the envelope calculation, the Food and Agriculture Organization reports that there were a million household poultry producers using data from around 2010, and other sources report a national inventory of around 33 million birds in 2005; that would suggest flock size per household of between 30 and 40. Flock size in this sample is somewhat larger, consistent with the oversampling of large producers, but not dramatically so.

13 The price employed to impute the value of chickens consumed is the mean sale price reported in the commune, using data from all households who report sales. The value of own-labor in poultry production is not estimated as a cost due to the absence of an appropriate wage measure; markets for hired agricultural labor are extremely thin in this context, and virtually no sample households report any meaningful use of external labor.

14 The correlation between the number of chickens reported owned in the census (in which all households in each sampled village were identified and their poultry flock size estimated, to enable construction of the sample, and the number reported owned in the comprehensive post-harvest baseline conducted approximately two to three months later is only about .4. Similarly, the correlation between the flock size reported in the post-harvest baseline and in the lean season baseline conducted six months later is only .3. Using the method described, we identify 55% of households as large producers, considerably higher than the sampling target of 40%.

15 There are some significant outliers in the reported mortality ratio, but even the median mortality ratio suggests very high losses on average: 55% for small producers, vis-a-vis 36% for small producers.

16 In general, the data around the probability of sales and price suggests relative stability in patterns of commercialization across seasons. At baseline, 65% of households report selling chickens in the reference period (the previous six months) in the main baseline survey conducted in March through June 2017; 70% report doing so in the lean season survey conducted in September 2017. At endline, the probability of sales varies between 72% and 75%. Prices are similarly stable across rounds.

17 We report the p-value from a regression in which the indicator of interest is regressed on a binary variable for assignment to SELEVER; the regression is weighted to take into account sampling probabilities and standard errors are clustered at the commune level.

18 The household covariates include household size, the age of the head of household, and the baseline flock size (number of mature birds).

19 Given that the sample was surveyed in part prior to COVID-related closures implemented in March and April 2020 and in part post-closures, we also can explore whether there are any substantial differences on average comparing across these periods. (80% of the sample was surveyed post-closures.) Pandemic-related closures in Burkina were relatively brief, as markets closed for only a month, and there was no systematic implementation of travel restrictions or curfews, especially in rural areas. Consistent with this fact, we do not observe any patterns that are suggestive of major adverse economic shocks for poultry production in our sample: the reported probability of any poultry revenue and prices are relatively stable (at 75% and $5 per bird, respectively), and the number of poultry reported sold and consumed increases post-closure. The increase in poultry sold in conjunction with a stable price is suggestive of higher demand; the most parsimonious explanation of this pattern may be that the recall period for households surveyed post-closures includes the Eid festival (observed in May 2020), a major date for poultry sales and consumption.

20 While we utilize ANCOVA as the primary specification of interest following the recommendation of McKenzie (2012) for variables in which autocorrelation is low, we also report difference-in-difference specifications in Table A2 in Appendix. The results are entirely consistent.

21 We also evaluated alternate measures of poultry mortality such as the mortality ratio, defined as the number of birds lost normalized by total flock size. While we see a pattern of negative effects on these other variables capturing mortality, the coefficients are not statistically significant.

22 At baseline, only 4% of households report revenue from egg sales, and these households report an average flock size of 122, a flock size corresponding to nearly the 99th percentile of the baseline distribution.

23 Similar propensity score matching techniques have been widely employed in evaluating interventions in developing countries; see for example Ninno and Dorosh (2003), Gilligan et al. (2009), de Brauw and Hoddinott (2011) and Asfaw et al. (2012).
