# Supplementary material for: How light is too light touch: The effect of a short training-based intervention on household poultry production in Burkina Faso
Source: J Dev Econ. 2022 Mar;155:102776. doi: 10.1016/j.jdeveco.2021.102776 (PMC8856925; doi:10.1016/j.jdeveco.2021.102776)
Supplement: MMC S1 — On-line appendix for how light is too light touch. [file mmc1.pdf]

Appendix: For online publication only

A1 Effects on poultry production by women

In addition to targeting increased household engagement in poultry production, the SE-LEVER intervention sought to enhance women's engagement in poultry production and increase their decision-making power. Gender-related programming was implemented in conjunction with nutrition programming providing behavioral change communication around diets and nutrition; the gender-related messages emphasized the importance of strengthening women's leadership within the community linked to poultry production, nutrition, and child health. These messages were delivered through training of community leaders; training of female leaders who would provide training to their members (including in women's poultry production associations); and training of champion husbands and model women who are particular leaders in gender equitable practices and who can train their peers.

As evident in the summary statistics provided in Table 1, clearly men report ownership of an overwhelming share of the household chicken flocks, while there is minimal reported ownership of chickens by women and jointly. Consistent with the low reported ownership levels for women, reported sales of poultry owned by women are also relatively rare: only 17% of women report any sales of their own poultry over the past six months, while 65% of men report sales of their own poultry. Evidence from qualitative work conducted as part of this evaluation largely suggests that household poultry flock are maintained in a single location, largely under the care of women (who care for their husband's poultry as well), but men maintain control over sales. Women use income from sales of their own poultry as a source of funds for emergencies or occasional discretionary expenditure, similar to the role that poultry sales play in poor households in general (Eissler et al., 2020).

To evaluate the effects of the intervention on gender-disaggregated measures of poultry production, we report in Table A4 the estimated treatment effects for primary outcomes of interest for poultry reported owned by men, women and jointly by multiple members of the household. Again, characteristics of the flock and associated poultry business decisions were reported separately by a male respondent (usually the household head) for poultry owned by the head as well as poultry owned jointly by multiple members of the household, and by a female respondent for poultry owned by the women of the household. The sample is consistent for each set of variables (male-owned, female-owned, and joint-owned) as all households report for all three categories, though they may report values of zero for any category. The only exceptions are household engagement in poultry

production (reported only for the household) and variables linked to egg revenue (reported only for the household).

In Panel A and Panel C we observe generally null (and often negative) effects for flock size, revenue and profits as reported for poultry owned by men and owned jointly by multiple members of the household. However, in Panel B we observe that for poultry owned by women, households in communities exposed to SELEVER report a significant increase in the number of mature birds, stock value, revenue and profits. Moreover, given the low mean level of poultry production reported for women, the effects are proportionately large: there is a 48% increase in the number of birds owned, a 45% increase in stock value, a 52% increase in revenue, and a 74% increase in profits. In absolute magnitude, this corresponds to increased profits of $1.40 for poultry owned by women over the six-month reference period.

If we compare the coefficients estimated in Panels B and C, the point estimates for poultry owned jointly reported in Panel C are negative, though not statistically significant, and generally of comparable magnitude to the point estimates for poultry owned by women. This is consistent with the overall null effect on household-level measures of poultry production previously reported. We also report in the bottom rows of Panels B and C p-values testing the equality of coefficients across the specifications estimated for poultry owned by men, women and couples. Given the noise in the estimated treatment effects, the hypothesis that the effects are equal for poultry owned by men and women cannot be rejected at conventional levels, but the hypothesis that the effects are equal for poultry owned jointly and poultry owned by women can be rejected.

One plausible interpretation of this pattern is that there has been a shift from jointly to solely (female) owned poultry within these households. Clearly, this shift cannot be directly substantiated given that we do not have retrospective data on ownership of particular chickens. Qualitative data suggests that women may designate chickens as jointly owned rather than solely owned as a mark of deference to their husbands or to avoid asserting their property rights over the asset directly (Eissler et al., 2020); if the gender component of the intervention then successfully shifts women's attitudes around the desirability of women's engagement in poultry production, they may be more willing to exert property rights over these previously jointly owned chickens.

Table A1: Cross-sectional Correlations: Household Characteristics and Poultry Revenue at Baseline

	Poultry revenue			
	(1)	(2)	(3)	(4)
Reports any shock	7.582**	1.342		
	(3.034)	(7.641)		
Enrollment rate under 18			-10.559	3.588*
			(13.184)	(2.108)
Obs.	608	1062	1062	608

Notes: The dependent variable is reported revenue from poultry sales over the preceding six months at baseline. The independent variables are a binary variable for the household reporting any adverse shock over the preceding year; and the enrollment rate of children under 18 reported in the household. (The regression for enrollment rate also controls for the number of boys among children under 18 in the household.) Standard errors are clustered at the commune level.

Table A2: Effect of SELEVER on Household Poultry Production:
Difference-in-difference Estimates

	(1)	(2)	(3)	(4)	(5)
		**Primary outcomes**			
	Stock value	Revenue	Profit		
SELEVER x Endline	-2.828	-10.643	-5.890		
	(6.433)	(7.547)	(10.643)		
SELEVER	11.165	9.032	4.014		
	(7.215)	(7.474)	(10.202)		
Endline	6.432	.021	-2.045		
	(4.853)	(4.573)	(7.067)		
Obs.	3338	3338	3338		
		**Robustness: Ancillary measures linked to primary outcomes**			
	Any poultry	Number mature birds	Any revenue	Any revenue eggs	Egg revenue
SELEVER x Endline	.006	4.698	.008	.007	.058
	(.017)	(2.965)	(.035)	(.020)	(.188)
SELEVER	.005	-.872	.024	-.013	-.066
	(.017)	(1.498)	(.036)	(.010)	(.078)
Endline	.023	-4.499*	.102***	.064***	.489***
	(.014)	(2.733)	(.027)	(.014)	(.113)
Obs.	3338	3338	3338	3338	3338

Note: This table reports results for the primary outcomes of interest. Using the panel dataset, the dependent variables are regressed on a binary variable for assignment to SELEVER, a binary variable for the endline round, and the interaction of the two; the specification includes demographic controls and is weighted to take into account baseline sampling probabilities, and standard errors are clustered at the commune level. The reference period for variables is generally the past six months. The variables of interest are as follows: in Panel A, the estimated value of the flock on the day of the survey, the total amount of revenue over the reference period (inclusive of the value of own-consumption), and the total amount of profits (again inclusive of the value of own-consumption), and in panel B, a binary variable for any poultry reported raised by the household over the reference period, the number of poultry owned on the day of the survey, whether the household reports any revenue from poultry sales over the reference period, whether the household reports any revenue from egg sales over the reference period, and the total amount of egg revenue over the reference period. Monetary variables are reported in real 2017 U.S. dollars. Asterisks indicate significance at the ten, five and one percent level.

Table A3: Household Expenditure and Crop Production

	(1)	(2)	(3)
	**Panel A: Household expenditure**		
	Total expenditure per capita	Food per capita	Non-food per capita
SELEVER	-.562	-5.948	7.793
	(19.762)	(6.499)	(18.049)
Mean control arm	260.842	64.454	193.714
Obs.	1662	1665	1662
	**Panel B: Crop-related outcomes**		
	Area cultivated	Total produced	Number crops
SELEVER	.560	-100.902	.342
	(.484)	(264.056)	(.354)
Obs.	1648	1640	1640

Note: This table reports results for household expenditure (in Panel A) and variables linked to crop production (in Panel B). The dependent variables are regressed on a binary variable for assignment to SELEVER, the baseline level of the dependent variable, and demographic controls; the regression is weighted to take into account baseline sampling probabilities, and standard errors are clustered at the commune level. Expenditure is reported in real 2017 CFA. Crop variables reported are area cultivated in hectares, total crop output produced in kilograms, and the number of crops. Asterisks indicate significance at the ten, five and one percent level.

Table A4: Effect of SELEVER on Household Poultry Production by Ownership Category

	(1)	(2)	(3)	(4)	(5)
	Number mature birds	Stock value	Any revenue	Revenue	Profit
			**Panel A: Poultry owned by men**		
SE LEVER	2.347	2.719	.031	1.112	-.988
	(1.829)	(7.000)	(.034)	(3.936)	(3.590)
Mean control arm	18.626	78.919	.645	40.14	30.515
Obs.	1669	1669	1669	1669	1669
			**Panel B: Poultry owned by women**		
SE LEVER	.996***	3.538***	.040	1.520**	1.385**
	(.345)	(1.240)	(.026)	(.688)	(.618)
p-value: $\beta^w = \beta^m$	0.336	0.977	0.810	0.795	0.920
Mean control arm	2.062	7.787	.147	2.911	1.94
Obs.	1669	1669	1669	1669	1669
			**Panel C: Poultry owned jointly**		
SE LEVER	-.459	-1.714	-.022	-1.913	-1.363*
	(.339)	(1.306)	(.016)	(1.441)	(.707)
p-value: $\beta^j = \beta^m$	0.107	0.492	0.341	0.654	0.266
p-value: $\beta^j = \beta^w$	.001	.001	.031	.001	.142
Mean control arm	1.194	5.012	.070	3.484	2.586
Obs.	1669	1669	1669	1669	1669

Note: This table reports results for the primary outcomes of interest as described in Table 5, as measured separately for poultry reported owned by men, owned by women, and owned jointly by the household. The specification is identical to that described in Table 5. Monetary variables are reported in real 2017 U.S. dollars. The p-value reported in Panels B and C correspond to a test of equality comparing across the coefficients in each column as estimated in Panel A vis-a-vis those estimated in Panels B or C. Asterisks indicate significance at the ten, five and one percent level.

Table A5: Selection into Treatment Participation

	(1)	(2)	(3)	(4)	(5)
	Reports benefits	Poultry training	Business training	Producers group	Any exposure
Any poultry	.00005	.0002*	.0001	.0002*	.0001
	(.00007)	(.0001)	(.0001)	(.00008)	(.0001)
Number mature birds	-.00005	-.00007	-.00009	.0001	.0001
	(.00008)	(.0002)	(.0001)	(.0002)	(.0003)
Stock value	.00009	.0001	.0001	-.00008	-.00002
	(.0001)	(.0002)	(.0001)	(.0002)	(.0002)
Any revenue	-.008	.072**	.057**	-.011	.073
	(.027)	(.031)	(.024)	(.031)	(.045)
Any revenue eggs	-.00002	-.0002**	-.0002***	-.00004	-.0003***
	(.00003)	(.00009)	(.00007)	(.00005)	(.00008)
Revenue	.004	-.006	-.016	-.002	.017
	(.016)	(.025)	(.021)	(.018)	(.032)
Egg revenue	-.104**	-.049	-.125	-.150***	-.233**
	(.041)	(.081)	(.108)	(.036)	(.099)
Profit	.012	.012	.051**	.023**	.058***
	(.010)	(.012)	(.023)	(.009)	(.020)
Head age	.0007	.0005	.0001	.002*	.0009
	(.0007)	(.001)	(.0009)	(.0008)	(.001)
Household size	.0007	.0005	.0001	.002*	.0009
	(.0007)	(.001)	(.0009)	(.0008)	(.001)
Head primary	.001	.004	.004	.0001	.003
	(.002)	(.004)	(.003)	(.003)	(.004)
Polygamous	.038	.026	.045	.023	.021
	(.031)	(.040)	(.033)	(.030)	(.050)
Polygamous	-.028	.010	.041	.009	.045
	(.020)	(.033)	(.027)	(.021)	(.037)
Obs.	1639	1639	1639	1639	1639
R^2	.007	.019	.039	.017	.026

Note: This table reports a series of regressions in which binary variables capturing participation in different dimensions of SELEVER programming are regressed on baseline covariates. The covariates are parallel to those reported in Table 1, and the dependent variables are parallel to those participation variables reported in Table 2. Asterisks indicate significance at the ten, five and one percent level.

Table A6: Effects of SELEVER: As Treated Analysis Using Propensity Scores

	(1)	(2)	(3)	(4)	(5)	(6)	(7)	(8)
	Stock value	Revenue	Profit	Any poultry	Number mature birds	Any revenue	Any egg	Egg revenue
**Panel A: Propensity scores using engagement in poultry**								
SELEVER	12.036	7.186	-.217	.001	4.015**	.064*	-.03	.05
	(9.304)	(9.014)	(13.921)	(.011)	(1.825)	(.039)	(.029)	(.3)
Obs.	1673	1673	1673	1673	1673	1673	1673	1673
R^2	.268	.177	.111	.108	.272	.071	.075	.053
**Panel B: Propensity scores using engagement in SELEVER**								
SELEVER (participation)	12.91	12.956	17.323	.02	6.964***	.096*	.036	.337
	(9.428)	(15.52)	(21.996)	(.019)	(2.816)	(.055)	(.037)	(.478)
Obs.	1673	1673	1673	1673	1673	1673	1673	1673
Mean control arm	95.286	48.168	36.363	.958	22.54	.749	.116	.867
St. dev. control arm	90.998	66.388	66.722	.200	20.859	.434	.321	3.327

Note: This table reports results for the primary outcomes of interest as described in Table 5. In Panel A, the subset of households in the treatment arm who report participation in SELEVER are compared to households in the control arm, including additional controls for the full set of baseline covariates analyzed in Table A5. In Panel B, a propensity score matching difference-in-difference specification is utilized to compare households who did and did not participate in SELEVER. Monetary variables are reported in real 2017 U.S. dollars. Asterisks indicate significance at the ten, five and one percent level.
